# Emergency right colectomy: which strategy when primary anastomosis is not feasible?

**DOI:** 10.1186/s13017-016-0073-6

**Published:** 2016-05-04

**Authors:** Hugo Teixeira Farinha, Emmanuel Melloul, Dieter Hahnloser, Nicolas Demartines, Martin Hübner

**Affiliations:** Department of Visceral Surgery, University Hospital of Lausanne (CHUV), Lausanne, 1011 Switzerland

## Abstract

**Background:**

Primary anastomosis is considered the standard strategy after right emergency colectomy. The present study aimed to evaluate alternative treatment strategies when primary anastomosis is not possible to prevent definitive ostomy.

**Methods:**

This retrospective study included all consecutive patients who underwent right emergency colectomy between July 2006 and June 2013. Demographics, surgical data, and postoperative outcomes were entered in an anonymized database. Comparative analysis was performed between patients with primary anastomosis (PA group) and those where alternative strategies were employed (no-PA group). Outcomes were 30 days complications rate and rate of bowel continuity restoration.

**Results:**

One hundred forty-eight patients (57 % male) with a median age of 65 years (15–96) were included. One hundred and sixteen patients underwent PA (78 %) and 32 were in the no-PA group (22 %). No-PA group patients had more comorbidities (Carlson comorbidity index >3: 98 % vs. 54, *p* < 0.001). Major complications rate (Dindo-Clavien III to IV) was 24 % in PA group, 88 % in no-PA group (*p* < 0.001). The 30-day mortality rate was 6 % (*n* = 7) in PA group versus 25 % (*n* = 8) in no-PA group (*p* = 0.004). Fourteen patients in the no-PA group had a split stoma and 18 had a two-staged procedure. Five patients had continuity restoration after initial split stoma (36 %) compared to 10 after a two-staged procedure (55 %; *p* = 0.265). Anastomotic leak occurred in 10 patients of the PA group (9 %) versus 0 in the no-PA group, where 15 out of 32 patients (47 %) had continuity restoration.

**Conclusion:**

Eighty percent of patients requiring emergency right colectomy were anastomosed primarily. For the remaining a two-staged procedure might facilitate bowel continuity restoration in the long-term.

## Background

Elective right colectomy entails a risk for postoperative complications and mortality around 22 and 1 % respectively [1, 2]. In the emergency setting, these rates grow up to 50 and 10 %, especially if risk factors are present. [3–5], Patient-related risk factors are age >70 years, male, malnutrition, ASA score >3, diabetes, tobacco smoking or immunosuppression. Procedure-related risk factors other than emergency include intra-operative blood transfusion, surgeon experience, operative duration or operations performed during night-shift [6–8].

Safety strategies are useful for emergency procedures if several risk factors are present. For *left*-sided emergency colonic resections, valuable options are creation of an end colostomy or primary anastomosis with diverting ileostomy [9, 10]. However, safety strategies have not been established for emergency *right*-sided resections. Resection with primary anastomosis remains the standard of care also in the emergency setting [11, 12]. Nevertheless, overall morbidity and mortality rates raise the question whether safer strategies are needed.

Therefore, the aim of the present study was to assess our institutional practice and outcome for emergency right colectomy and to evaluate alternative treatment to primary anastomosis and if definitive ostomy rate can be reduced.

## Methods

### Patients

This retrospective audit analysis included all consecutive patients who underwent a right-sided emergency colectomy from July 2006 to June 2013 in the department of visceral surgery, in Lausanne University Hospital. Right emergency colectomy included formal right colectomy including resection of up to 20 cm of small bowel. Transverse colic resections or extended right colonic resections were excluded. Emergency operation was defined as being performed during an unplanned hospital admission. Indication for surgery was given by the surgeon on call. Surgeries were performed by a board certified surgeon. Although this is a retrospective study, Swiss law demands that we submit the project to an Ethics Committee. The study was approved by the local Ethics Committee (University of Lausanne, Switzerland).

### Data collection

Demographics and risk factors as well as outcome measures were defined *a priori* and entered in an anonymized database. All data were collected retrospectively after the last included patient was operated.

Demographic data and patients’ co-morbidities (diabetes, obesity, chronic renal failure, cirrhosis, cardiopathy, tobacco smoking or immunosuppressive drugs consumption including corticoids, anti-TNF and chemotherapy) were included in the database. Co-morbidities and patient preoperative health were prospectively graded using the Charlson co-morbidity Index and *American Society of Anesthesiology* (ASA) score [13]. Surgical data included operative time, blood loss, as well as intraoperative vasopressor requirements (Noradrenalin >10ug/min intravenously) or surgeon’s expertise (junior or senior consultant)[14–16]. Junior staff are within 5 years after surgical graduation. Senior consultants have completed surgical training at least 5 years ago and/or have done a fellowship.

The retrospective cohort was divided into two groups. The first group included all patients with non-protected primary anastomosis (PA group) at the time of the intervention. All types of anastomotic techniques (end-to-end, side-to-end, side-to-side; hand-sewn or mechanical) were included. The second group without primary anastomosis (No-PA group) included patients who received either primary split stoma or who had just resection without primary anastomosis and a planned second look (so called two-staged procedure). Split stoma was defined by exteriorisation of both ends of the bowel through the same hole. The proximal end formed the functioning stoma and with faeces pass. The distal end of bowel was brought out through the abdominal wall and formed a non-functioning stoma. Split stoma procedure may permit a bowel continuity restoration without performing a laparotomy.

### Outcomes

Overall postoperative 30-day complications rate including mortality and the rate of bowel continuity restoration were the main outcomes. Complications were classified according to the Clavien-Dindo grading of surgical complications [17].The complication with the highest severity for each patient was considered for the analysis.

Other outcomes included length of intensive care unit (ICU) stay (days), length of hospital stay (days), destination after discharge (home or rehabilitation) and time to stoma reversal (months).

The study included all cases of stoma reversal after split stoma at fist intention, or after split stoma performed during a planned second-look following a two-stage procedure. Reasons not to close the stoma were entered in the database.

### Statistical analysis

Descriptive statistics for categorical variables were reported as frequency (%), while continuous variables were reported as median (interquartile range: IQR). Chi-square was used for comparison of categorical variables and the Wilcoxon test for continuous data. All statistical tests were two-sided and a level of 0.05 was used to indicate statistical significance. Data analyses were performed using SPSS Inc. released 2012.for Mac, Version 21.0. Chicago: SPSS Inc.

## Results

### Patients

**Fig. 1:**
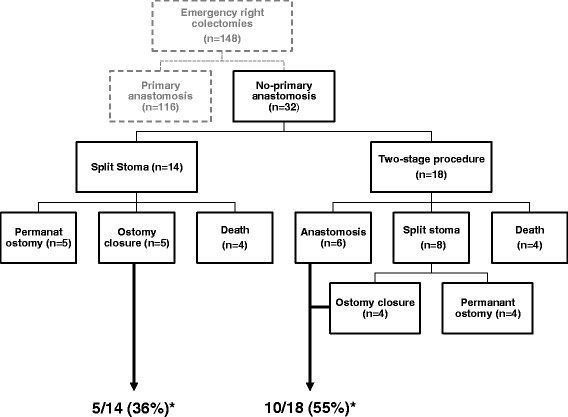
Population flow chart. *Percentage of colic continuity restoration after Split stoma and Two-stage procedure respectively

One hundred and forty-eight patients underwent emergency right-sided colectomy during the study period. Primary anastomosis (PA group) was performed in 116 (78 %) patients. Of the remaining 32 patients (=no-PA group), 14 (9.5 %) received a primary split stoma, while 18 (12.5 %) had a two-stage procedure (Fig. 1).

Demographic information for the two comparative groups are displayed in Table 1. Patients in the PA group were younger and had a lower BMI and ASA score as well as less co-morbidities.

**Table 1:** Comparison between PA group and no-PA group, Demographic Data

	PA group (*n* = 116)	No-PA group (*n* = 32)	*P* value
Age (range)	62 (15-90)	68 (27-94)	0.004
Sex ratio, (M:F)	67:49	18:14	1.000
Body mass index >25 (Kg/m2)	43 (37 %)	20 (63 %)	0.023
ASA grade III-IV, n (%)	67 (58 %)	29 (91 %)	<0.001
Charlson comorbidity index >3	67 (58 %)	30 (94 %)	<0.001
Comorbidity			
Diabetes, n (%)	20 (17 %)	6 (19 %)	0.798
Cardiopathy, n (%)	28 (24 %)	17 (53 %)	0.002
Tobacco smoking, n (%)	35 (30 %)	12 (38 %)	0.520
Immunosuppression, n (%)	12 (10 %)	10 (31 %)	0.009
Surgical indication			
Mechanical obstruction, n (%)	48 (41 %)	4 (13 %)	0.004
Perforation, n (%)	29 (25 %)	13 (41 %)	0.129
Hemorrhage, n (%)	16 (14 %)	1 (3 %)	0.173
Ischemia, n (%)	14 (12 %)	11 (34 %)	0.006
Other, n (%)	9	3	
Operator			0.551
Junior Consultant, n (%)	57 (49 %)	18 (56 %)	
Senior Consultant, n (%)	59 (51 %)	14 (44 %)	
Surgery time			0.831
Nightshift, n (%)	36 (31 %)	9 (28 %)	
Intraoperative Noradrenalin >10ug/min	36 (30 %)	30 (95 %)	<0.001
Surgical approach			0.202
Open, n (%)	108 (93 %)	32 (100 %)	

### Surgical data 

The most frequent indication for emergency right colectomy was mechanical obstruction (*n* = 52). Seventy-one percent (*n* = 37) overall obstructions were due to malignant obstruction. Other causes of obstruction were ileus due to adhesions (*n* = 7), obstruction due to inflammatory disease (*n* = 4), caecal volvulus (*n* = 3), and one hernia. All obstructions in the no-PA group were due to malignant lesions. In the no-PA group, perforation and ischemia were the prominent underlying pathologies. Obstruction and ischemia were indications that significantly differ between the groups. Patients in the PA group received significantly less intraoperative Noradrenalin than the others during surgery. Estimated blood losses (ml) were comparable between both groups Table 1.

There was no difference in the surgical management regarding surgeon expertise or between day and nightshift. Out of 116 anastomosis in the PA group, 28 (25 %) were stapled. The median operation time was 166 min (55–400 min) in PA group versus 107 min (47–338 min) for no-PA group (*p* = 0.003) Table 1.

### Outcomes

There were significantly more major complications (Clavien-Dindo III-IV) including more bleeding requiring transfusion in no-PA compared to PA group. The rate of surgical site infection (SSI) or postoperative ileus was similar between the two groups. Overall, the most common complication was SSI in both groups. Anastomotic leak occurred in 10 patients of the PA group (9 %) versus 0 in the no-PA group, where 15 out of 32 patients (47 %) had continuity restoration. All leaks were managed by reexploration and reanastomosis. Mortality occurred in 7 cases in PA group and in 8 cases in no-primary anastomosis group (6 versus 25 %) Table 2.

**Table 2:** Comparison between PA group and no-PA group; 30d complications and outcomes

	PA group (*n* = 116)	No-PA group (*n* = 32)	*P* value
30d complications			
overall	72 (62 %)	32 (100 %)	<0.001
III-IV, n (%)	28 (24 %)	28 (88 %)	<0.001
V, n (%)	7 (6 %)	8 (25 %)	0.004
Surgical site infection, n (%)	27 (23 %)	10 (31 %)	0.364
Postoperative ileus, n (%)	19 (16 %)	4 (13 %)	0.784
Need for Transfusion, n (%)	13 (11 %)	12 (38 %)	0.001
Anastomotic leak, n (%)	10 (9 %)	0*	
ICU stay in days (SD)	5 (16)	10 (13)	0.063
LOS in days (SD)	12 (21)	18 (24)	0.163
Discharge home	76 (66 %)	5 (16 %)	<0.001

*15/32 patients had anastomosis

In the PA group, 3 patients died of multiple organ failure (MOF) associated with a sceptic shock of abdominal origin, one caused by an anastomotic leak (14 %). Three patients died of respiratory failure, one caused by pleural effusion, one caused by pulmonary embolism and one caused by bronchoscopic aspiration. One patient died of hemorrhagic shock of colic origin.

In the No-PA group, 4 out of 14 patients died after split stoma (29 %) and 4 out of 18 patients died after two-stage procedure (22 %) before the planned second look. Reasons for postoperative death were multi organ failure (MOF) for 6 patients all caused by a septic shock of abdominal origin. One patient died of postoperative hemorrhagic shock of colic origin and one after ruptured aortic aneurysm.

Mean ICU stay was not different between the two groups while mean of hospital stay was significantly higher in the no-PA group. More patients were able to go home after discharge in the PA group without transfer to another hospital or to a rehabilitation centre Table 2.

Fourteen patients in the no-PA group had a split stoma and 18 had a two-stage procedure with a planned second look. Median time for planned second look was 48 hours (24–96). These two populations were comparable regarding demographics, co morbidities or surgical indications Table 3.

**Table 3:** Comparison between patient of the no-PA group who underwent split stoma or two stage procedure

	Split stoma (*n* = 14)	Two stage (*n* = 18)	*P* value
Age (range)	68 (27-88)	70 (34-94)	0.912
Sex ratio, (M:F)	7:7	11:7	0.532
Body mass index >25 (Kg/m2)	9 (64 %)	11 (61 %)	0.854
ASA grade			0.400
I-II, n (%)	2 (14 %)	1 (6 %)	
III-IV, n (%)	12 (86 %)	17 (95 %)	
Charlson comorbidity index >3	13 (93 %)	17 (94 %)	0.854
Comorbidity			
Diabetes, n (%)	2 (14 %)	4 (22 %)	0.568
Cardiopathy, n (%)	9 (64 %)	8 (44 %)	0.265
Tobacco smoking, n (%)	5 (36 %)	7 (39 %)	0.854
Immunosuppression, n (%)	4 (29 %)	6 (33 %)	0.773
Surgical indication			
Mechanical obstruction, n (%)	2 (14 %)	2 (11 %)	0.787
Perforation, n (%)	5 (36 %)	8 (44 %)	0.618
Hemorrhage, n (%)	0	1 (6 %)	0.370
Ischemia, n (%)	5 (36 %)	6 (33 %)	0.888
Other, n (%)	2	1	
Operator			0.127
Junior Consultant, n (%)	10 (71 %)	8 (44 %)	
Senior Consultant, n (%)	4 (29 %)	10 (56 %)	
Surgery time			0.960
Nightshift, n (%)	4 (29 %)	5 (28 %)	
Intraoperative Noradrenalin >10ug/min	12 (86 %)	18 (100 %)	0.098

Five out of 14 patients had an ostomy closure after split stoma within a median of 6 days (4–120). Of those one patient had an ostomy closure during the same hospitalisation, and 4 were readmitted for ostomy closure with a median hospital stay of 16 days (13–39). After ostomy closure, one patient had an anastomotic leak and needed a reoperation and refection of the anastomosis during the same hospitalisation. Four patients died before ostomy closure and 5 were not deemed eligible for another operation for medical reasons.

Six out of 18 patients who underwent a two-stage procedure had an anastomosis performed during the second look except for one patient who needed a complementary colic resection of 5 cm during the second look and anastomosis was performed at third look. One of those 6 patients had a leakage and needed a reoperation and anastomosis refection during the same hospitalisation. Eight patients had a split stoma during the second look. Two patients needed a complementary colic resection of 4 and 10 cm during second look. Four of the 8 patients who had a split stoma after a second look had an ostomy closure in a third time within a median of 63 days (57–67).

Regarding patients in the No-PA group, more patients had continuity restoration after two-stage procedure compared after split stoma 10 vs. 5, but this numbers were no statistically significant (*p* = 0.265) Fig. 1.

## Discussion

Primary anastomosis was performed in most patients after emergency right colectomy. Due to retrospective data analyse and obvious differences between patients from PA group and from no-PA group are incomparable. Two bailout options were applied in patients at high risk: split stoma confection and two-staged procedure with delayed ostomy or anastomosis. Our results suggest that a two-stage strategy might help to reduce permanent ostomy rate.

In accordance with the current literature[18], primary anastomosis was performed in 80 % of patients in this study Anastomotic leak rate was 9 % which was slightly higher than in the literature (4–6 %) probably because of increased co-morbidities, particularly more cardiac disease (30 % in this present study vs. 15 to 20 % in other studies) and higher ASA score 64 % III-IV vs. 40–50 %)[4, 18, 19]. None of these leaks resulted in death.

As expected, patients in the group with no primary anastomosis were significantly sicker and older. Furthermore, intraoperative risk factors and aetiologies differed significantly. All of these parameters have arguably influenced on surgical decision-making. Unfortunately, due to the retrospective nature of this study, it remains unclear which risk factors influenced surgical strategy most. Of note, the choice of surgical strategy was not influenced by surgeon’s experience in our present series as suggested by other reports [4].

Two main factors may probably have influenced the decision-making in the present study, both having a major impact on blood supply of an eventual anastomosis and hence its perceived safety. High intraoperative vasopressor requirements (>10ug/min of noradrenalin/min) and colic ischemia were more common in the no-PA group. In accordance, safety strategies were liberally employed on a case-by-case basis. It would be interesting to analyze the pathway of decision-making but due to emergency and retrospective analysis we could not do that. Interestingly, primary anastomosis was performed with good results even in case of tumour obstruction with proximal bowel dilatation. Surgeon’s experience or dayshifts did not play any role on strategy decision or on postoperative complications. Even when bailout procedures were performed and primary anastomosis was avoided, outcomes were disappointing in the high-risk patients group with an overall morbidity of 100 % and a mortality of 25 %. Other groups reported similar results underlining the overwhelming impact of the concomitant metabolic stress response do to preoperative comorbidities, emergent surgery and hemodynamic instability during anaesthesia [20]. A surgical safety strategy can only aim to obtain local control with low surgical morbidity; avoiding a high-risk anastomosis can certainly play a role in this concept. Further, surgical aggression should be reduced to a minimum in the context of an overshooting systemic inflammatory response; this can be achieved by primary resection, open abdomen and second look once the patient has been stabilized [21]. Nevertheless, outcomes remain dismal and early and aggressive reanimation at the intensive care unit is arguably as decisive with regards to outcomes as surgery[22]. The surgeon's decision seems to be adequate when a primary anastomosis is chosen. Mortality and morbidity rate are low and comparable to series in the literature [1, 23].

One of the most interesting finding of this study was the difference in permanent ostomy rate within the group of 18 patients who had a two-staged procedure. After right colectomy, the surgeon chooses to perform a second-look 2–4 days later either because the patient was deemed to be unstable to continue the intervention or the surgeon wanted to reassess the viability of the remaining intestine (usually in an ischemic context) before restoring bowel continuity. By applying this strategy more continuity restoration was done compared to the group with primary split soma (10/18 vs. 5/14; *p* = 0.265). The patients in split stoma groups and two-stage procedure do not differ in their co-morbidities, the patients seem to benefit from two-stage procedure. However, this comparison is too small to draw final conclusions, but a two-staged procedure appears to be a valid approach and a bail out option for selected high-risk patients. A two-staged procedure with planed second-look allows a short first surgery (“just” the resection or damage control), minimizes surgical trauma and allows for early intensive care. If the evolution is favourable, some to these patients can still benefit from an anastomosis at planned second or third look. However, a two-staged procedure implies easy access to operating rooms, which could be difficult to achieve in some centres.

This study also shows that junior surgeons more often performed split stoma than two stage procedures. Comparison with senior surgeons was not significant, but a trend can not be denied. Unfortunately, we did not record reasons that drove junior consultants to opt for split stoma.

The present study is limited by its retrospective design. Furthermore, results from a single centre cannot be generalized by principle. However, this audit might help in certain situations with the decision to anastomose or not. It definitely warrants a prospective trial. Only large datasets could help to overcome limitations of heterogeneity and low power.

## Conclusions

In conclusion, primary anastomosis was performed in 80 % of patients undergoing emergency right-sided colectomy. In patients considered more fragile (e.g. patients with heart disease, immunocompromised patients, hemodynamically unstable or with ischemic colic lesions) and where the surgeon initially does not anastomose, a two-staged procedure with a second look might facilitate continuity restoration in the long-term.

## Abbreviations

ASA: American Society of Anaesthesiologists
ICU: Intensive Care Unit
PA: Primary anastomosis
SSI: Surgical Site Infection
